# *β*-Nicotinamide Mononucleotide Promotes Cell Proliferation and Hair Growth by Reducing Oxidative Stress

**DOI:** 10.3390/molecules29040798

**Published:** 2024-02-08

**Authors:** Chuntao Xu, Jiawei Dai, Hongxia Ai, Weian Du, Hongbing Ji

**Affiliations:** 1 School of Chemistry and Chemical Engineering, Guangxi University, Nanning 530004, China; chuntao@zspt.edu.cn (C.X.); 2114402022@st.gxu.edu.cn (J.D.); 2 Guangdong Institute of Modern Agricultural Equipment, Guangzhou 510145, China; 3 Guangdong Homy Genetics Ltd., Foshan 528000, China; duwa168@163.com; 4 State Key Laboratory Breeding Base of Green-Chemical Synthesis Technology, Institute of Green Petroleum Processing and Light Hydrocarbon, College of Chemical Engineering, Zhejiang University of Technology, Hangzhou 310014, China

**Keywords:** β-Nicotinamide mononucleotide (NMN), dermal papilla cell, minoxidil, androgenetic alopecia

## Abstract

β-Nicotinamide mononucleotide (NMN) has shown promising effects on intestinal health, and it is extensively applied as an anti-aging and Alzheimer’s disease therapeutic, due to its medicinal properties. The effects of NMN on the growth of mouse hair were observed after hair removal. The results indicated that NMN can reverse the state of hair follicle atrophy, hair thinning, and hair sparsity induced by dihydrotestosterone (DHT), compared to that of minoxidil. In addition, the action mechanisms of NMN promoting hair growth in cultured human dermal papilla cells (HDPCs) treated with DHT were investigated in detail. The incubation of HDPCs with DHT led to a decrease in cell viability and the release of inflammatory mediators, including interleukin-6 (IL-6), interleukin-1Beta (IL-1β) and tumor necrosis factor Alpha (TNF-α). It was found that NMN can significantly lower the release of inflammatory factors induced by DHT in HDPCs. HDPCs cells are protected from oxidative stress damage by NMN, which inhibits the NF-κB p65 inflammatory signaling pathway. Moreover, the levels of androgen receptor (AR), dickkopf-1 (DKK-1), and β-catenin in the HDPCs were assessed using PCR, indicating that NMN can significantly enhance the expression of VEGF, reduced IL-6 levels and suppress the expression of AR and DKK-1, and notably increase β-catenin expression in DHT-induced HDPCs.

## 1. Introduction

Hair is an important part of human body, but hair loss has been a global public health problem that is mainly related to the aging process. Various factors can cause hair loss, including mental stress, disease, autoimmune diseases, and drug treatments [1,2]. The main cause of hair loss, especially in men, is attributed to a decrease in male hormones. Male hair loss mainly includes two types: androgenetic hair loss and paternal pattern hair loss [3]. Individuals genetically predisposed to androgenetic alopecia (AGA) also experience symptoms related to androgens. Hair loss has not attracted considerable attention in daily life, but it can significantly decrease patient’s quality of life [4,5,6,7]. Therefore, knowledge of how to decrease and repair hair loss has become even urgent. Various methods, such as laser and drug therapy and surgical intervention, have been used for the treatment of hair loss [8]. Minoxidil and finasteride have been effective drugs to promote hair growth in most hair loss patients with androgenetic alopecia [9,10]. However, these drugs may cause adverse side effects such as erectile dysfunction, fetal malformation, and hirsutism. To address these issues, researchers have been dedicated to finding new bioactive compounds and drugs from natural extracts and organisms that can stimulate hair growth [11]. NMN is a potential functional substance with a wide range of biological sources, which is relatively high in some vegetables such as broccoli, avocado, cucumber, and cabbage [12,13]. The cyclical growth of hair is regulated by paracrine and autocrine factors, including growth-promoting factors, such as vascular endothelial growth factor (VEGF), insulin-like growth factor-1 (IGF-1), fibroblast growth factor (FGF), keratinocyte growth factor (KGF), and growth inhibitors such as dickkopf 1 (DKK-1), Interleukin-6 (IL-6), and androgen receptor (AR) [14]. However, the stimulation of hair growth is facilitated by VEGF, which can promote angiogenesis and vasculogenesis, thereby enhancing nutrient delivery to hair follicles. This process is achieved through an increase in the diameter of the follicle base. Previous studies have shown that VEGF expression is significantly lower in alopecia follicles compared to normal follicles [15]. In comparison to non-balding HDPCs, IL-6 is upregulated in balding human HDPCs. It can also inhibit keratinocyte (KCs) proliferation and hampers hair shaft elongation [12,16]. The characteristic feature of androgenetic alopecia (AGA) was found to significantly decrease in size of hair follicles during repeated hair cycles, resulting in the production of thin, non-pigmented vellus hairs with a shorter anagen phase [17]. Recent results indicate that a correlation between AGA and non-AGA was observed. In addition, high expression levels of AR (androgen receptor) and AR-5α-DHT complexes can effectively interact with genes to decrease in size of hair follicles, leading to hair loss in AGA [18]. Therefore, the expression level of AR is an effective factor in hair loss associated with AGA. DKK-1, an inducer of catagen, which plays a role in regulating hair follicle cycling [19]. Human balding HDPCs exhibit upregulation of DKK-1 as a response to DHT [20]. DKK-1 disrupts Wnt signaling and interferes with hair growth. Wnt/β-catenin has been identified as a crucial factor in hair growth, encompassing hair follicle morphology, initiation of hair follicle growth, and stimulation of hair regeneration [21].

NMN serves as a crucial intermediate in the synthesis of nicotinamide adenine dinucleotide (NAD+). It is an intermediate in the NAD+ salvage synthesis pathway, formed by the reaction of nicotinamide (NAM) with 5-phosphoribose-1-pyrophosphate (PRPP) under the catalysis of nicotinamide phosphate ribose transfer (NAMPT). Extracellular NMN needs to be converted into nicotinamide nucleoside (NR) through dephosphorylation reaction to enter the cell, and then dephosphorylated under the catalysis of nicotinamide nucleoside kinase (NRK) to generate NMN, which is generated by reacting with ATP under the catalysis of nicotinamide mononucleotide adenosine transferase (NMNAT) NAD+ [22]. NAD+ is a nucleotide with significant biological activity, primarily located in cellular mitochondria. NMN itself consists of two isomers, namely the α-isomer and the β-isomer, which are derivatives of vitamins B [23]. It is the β-isomer that functions as the active form of NMN and can be found in various organisms. Numerous studies have shown the advantageous effects of NMN, which are reflected in intestinal health, as well as its potential in combating cardiovascular and cerebrovascular diseases, neurodegenerative disorders, and the process of aging [24]. Furthermore, its potential applications are mainly in preventing obesity and diabetes by regulating endocrine functions and increasing insulin secretion. Experimental studies also indicate that NMN has the ability to delay aging, enhance skin whiteness, prevent various health problems, boost energy levels, improve sleep quality, facilitate DNA repair, eliminate alcohol toxins, safeguard the liver, and maintain visual acuity [25,26]. However, the effects of NMN on hair growth promotion and stimulation of HDPC proliferation have not been very clear yet.

This study aims to evaluate the effects of NMN on hair growth, as well as to explore its action mechanism. The proper development and regeneration of hair follicles rely on the influence of HDPCs in embryonic hair follicles. In order to create an androgen alopecia model, cultured HDPCs were stimulated with DHT. The assessment of expression levels of regulatory factors associated with hair growth was conducted as the evaluation metric.

## 2. Results

### 2.1. Effects of NMN on Cell Proliferation

**Figure 1:**
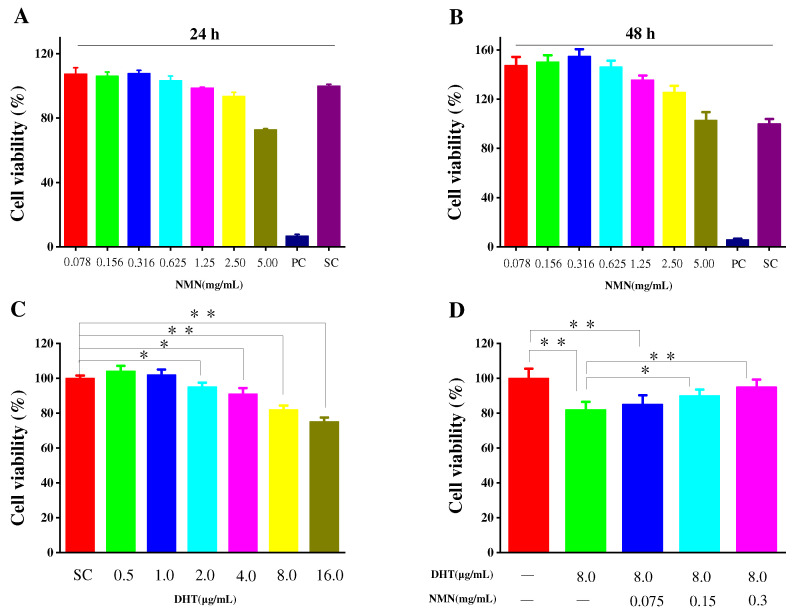
Cell viability of HDPCs. (**A**,**B**) The HDPCs activity in response to NMN at 24 h and 48 h, respectively. (**C**) The effect of DHT concentration on the activity of HDPCs. (**D**) NMN repair ability of DHT induced damaged HDPCs. Cell viability was analyzed using the MTT assay. PC (positive control group): 10% DMSO in 200 μL of culture medium, SC (solvent control group): 200 μL culture medium. Data are presented as the mean ± SD (*n* = 3). The significance levels are expressed as: *p*-value < 0.05 as *, and *p*-value < 0.01 as **.

The secretion of cytokines by HDPCs plays an important role in the hair growth cycle. To assess the potential toxicity of NMN on HDPCs, MTT assays were conducted at different concentrations for 24 and 48 h, respectively, which are used to determine the appropriate amount of NMN for subsequent experiments. As shown in Figure 1A, HDPCs exhibited enhanced proliferation when stimulated with NMN concentrations below 0.625 mg/mL. This proliferation decreased when NMN concentrations were 1.25 mg/mL. However, cell viability above 90% has no significant impact on the subsequent experimental results. HDPCs displayed a proliferation rate of 93.59% at an NMN concentration of 2.5 mg/mL. According to the MTT results, it is believed that NMN based on HDPCs did not exhibit significant cytotoxicity within the concentration range of 2.5 mg/mL. After 48 h of cultivation, varying degrees of HDPC proliferation were observed at different NMN concentrations. The highest cell proliferation was achieved at 0.316 mg/mL NMN, as shown in Figure 1B. In addition, the effects of DHT on HDPC activity was also examined, implying that HDPC activity gradually decreased with the increasing DHT concentration. As depicted in Figure 1C, when the NMN concentrations were above 8 μg/mL, the activity of HDPCs was influenced by NMN concentration, indicating varying degrees of cellular damage. Furthermore, when HDPCs were exposed to DHT and treated with different doses of NMN, it was observed that NMN could repair the damage caused by DHT. The repair ability of NMN significantly increased with rising NMN concentrations, as illustrated in Figure 1D.

### 2.2. Induction of Hair Growth in Resting Mice by NMN

**Figure 2:**
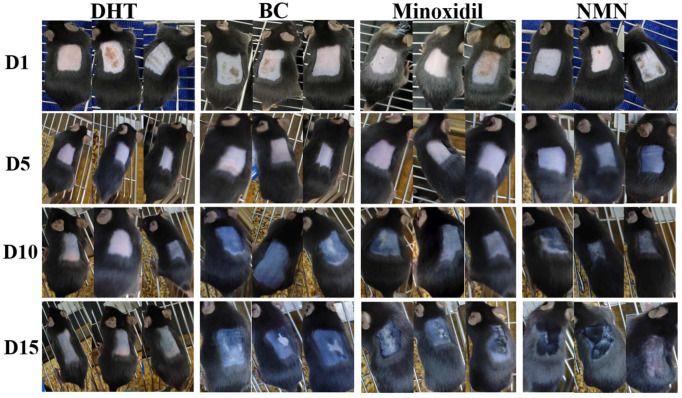
Induction of the anagen phase by NMN in depilated C57BL/6 mice. Photographs were taken of the dorsal skins at 1, 5, 10, and 15 days after depilation, respectively. The DHT groups are the negative control groups (5% DHT *m*/*v*), BC groups: blank groups, Minoxidil groups: positive controls (5% minoxidil *v*/*v*), and NMN groups: 1%NMN, *m*/*v*.

**Figure 3:**
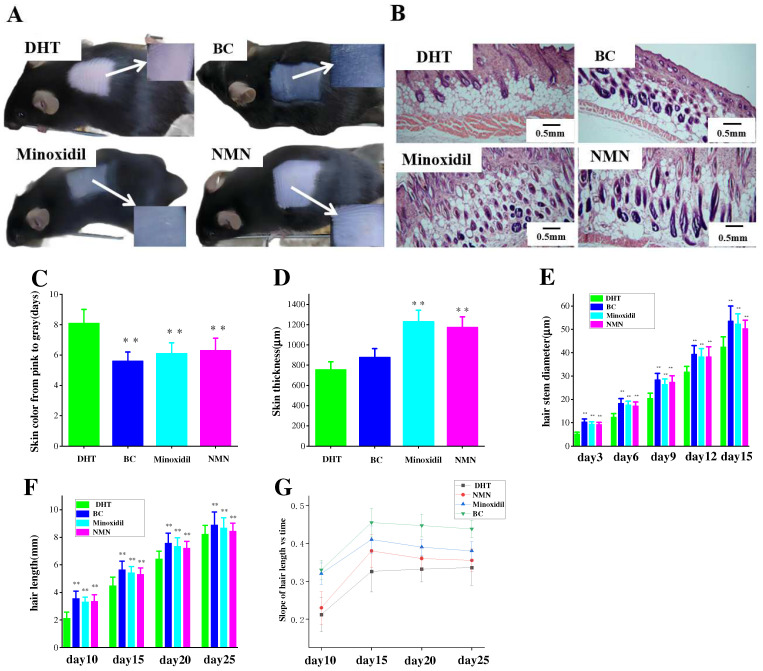
The effects of hair regrowth treatments on the skin of male C57BL/6 mice with Hematoxylin and Eosin (H & E) staining. (**A**) Comparison of skin color on the fifth day after hair removal in mice. (**B**) H & E staining was conducted along the long axis of each dorsal skin section for each group. (**C**) The time required for the skin color of each group to change from pink to gray. (**D**) Average skin thickness across the groups. (**E**–**G**) Comparison of the diameter and hair length of dried hair in mice at different time periods after hair removal. The results represent the average of three parallel experiments. ** *p* < 0.01, compared to the control group.

The effects of NMN on hair regeneration were assessed in C57BL/6 mice. Based on the guidelines for animal care and usage, C57BL/6 mice were acclimated to the experimental setting for one week, starting at the age of 5 weeks. Prior to hair removal for two weeks, the mice were injected with DHT and NMN were use as designated drugs. The hair growth of the NMN-treated groups was compared to those of the DHT-treated groups (negative groups), the groups treated with 5% minoxidil (positive groups), and the BC groups (blank groups). The results showed that at 5 days after hair removal, most of the skin burns resulting from the initial modeling stage had healed (Figure 2). In addition, the skins of mice in the minoxidil-treated, NMN-treated, and BC groups began to darken (Figure 3A), implying forthcoming hair growth. At 10 days, the mice in the positive groups, BC groups, and the NMN groups displayed short hair growth, but the skins in the control groups started to darken. The control groups exhibited little hair growth. For the experimental conclusions at 5 days, the mice in the positive groups, BC groups, and the NMN groups exhibited significant hair growth, concealing the previously shaved skin. Therefore, the skins of the control groups continued to exhibit minimal hair growth.

### 2.3. The Effect of NMN on Hair Growth Improvement

At 5 days after hair removal, a specific area of the mouse skins was subjected to Hematoxylin and Eosin (H & E) staining. Comparative analysis revealed a significant disparity between the groups injected with NMN and minoxidil, respectively, regarding their ability to stimulate the transition of hair follicles from the telogen to the anagen phase (Figure 3B). As shown in Figure 3C, both the NMN and minoxidil groups exhibited a faster darkening for the skin color, compared to the control group. A measurement of skin thickness was displayed in Figure 3D, indicating that the NMN groups exhibited greater skin thickness in comparison to the control groups and demonstrated similar thickness to the minoxidil groups. The BC groups also had thicker skin in comparison to the control groups, which were in accordance with the corresponding hair expression in the mice. In addition, an evaluation of the direct and growth rate of hair was carried out (Figure 3E,F). The results showed that the presence of DHT prolonged the growth rate of hair and induced a trend of hair shaft thinning, but NMN effectively ameliorated the conditions.

### 2.4. Enhancement of Hair Follicle Growth by NMN

**Figure 4:**
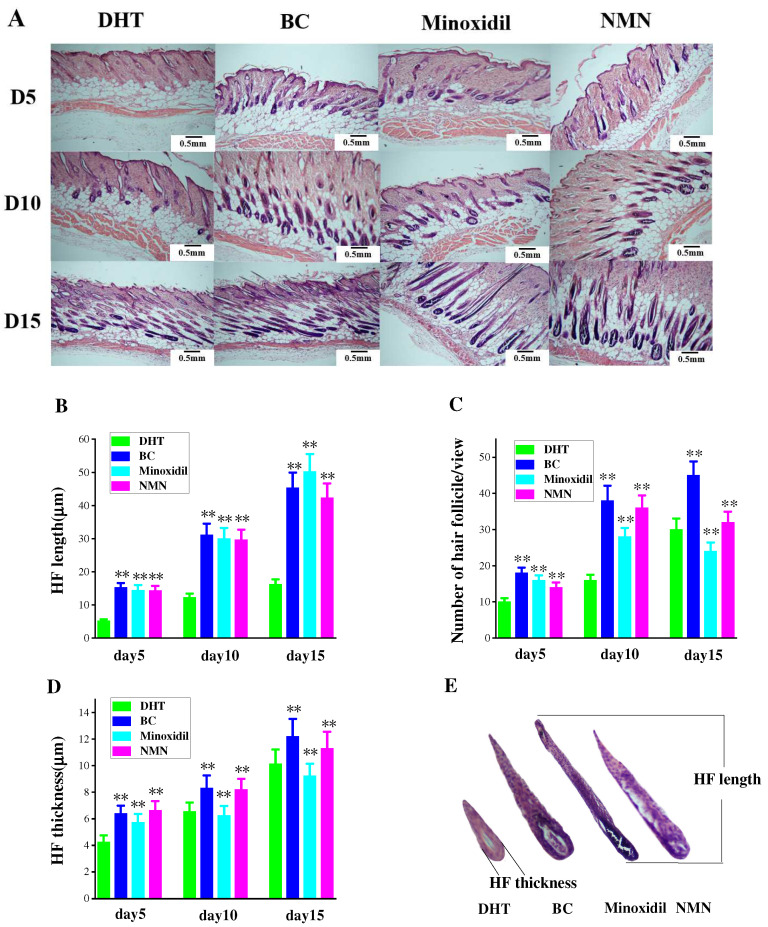
The growth and development of hair follicles. (**A**) Evaluated the growth of hair follicles at different stages using H & E staining. (**B**) Changes in hair follicle length in mice after hair removal. (**C**) The increased in the number of hair follicles in mice over time after hair removal. (**D**) Changes in hair follicle thickness over time after mice hair removal. (**E**) On the tenth day after hair removal, the size of hair follicles in each group. The results represent the average of three parallel experiments. ** *p* < 0.01, compared to the DHT group.

Observation of hair follicle changes in experimental mice was conducted through H & E staining of tissue slices at various time intervals. Figure 4A reveals the initial growth of hair follicles on the skin’s surface, followed by a gradual descent. Comparatively, the rate of downward movement was slower in the DHT group than in both the minoxidil and NMN groups. Additionally, the coloration of hair follicles in the NMN and minoxidil groups appeared darker in comparison to the DHT group. Figure 4C further demonstrates that the number of hair follicles in the NMN and minoxidil groups significantly exceeded that in the control group. Furthermore, the hair follicle size, encompassing length and thickness, depicted in Figure 4B,D, was notably smaller in the DHT group than in either the NMN or minoxidil groups.

### 2.5. Effects of NMN on VEGF Expression in HDPCs

**Figure 5:**
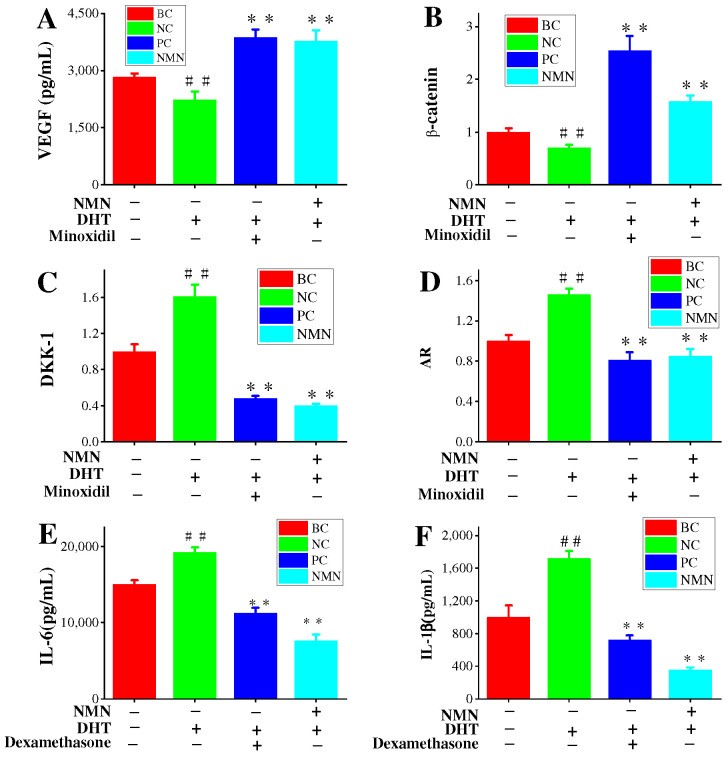
Effects of NMN on the regulatory signals of HDPCs. (**A**) The contents of VEGF expression in HDPCs. (**B**,**C**) The related expression of β-catenin/Wnt signaling pathway. (**D**) Expression of biomarkers for DHT induced hair loss. (**E**,**F**) Content of inflammatory factors IL-6 and IL-1. Dexamethasone was used as a positive control for inflammatory factors such as IL-6 and IL-1β. Minoxidil was used as a positive control for VEGF, DKK1, AR β-catenin gene. Statistical analysis was conducted using the *t*-test, with the BC groups as the reference. Significance levels are expressed as: *p*-value < 0.01 as ##. When compared with the NC groups, significance levels are expressed as: *p*-value < 0.01 as **.

Previous results have confirmed the promotion of hair growth by VEGF. Additionally, VEGF has played a significant role in the treatment of AGA with minoxidil [27,28]. Therefore, the effects of NMN on VEGF expression in HDPC cultures are of great interest. As presented in Figure 5A, compared to the BC groups, the VEGF content in the control groups was significantly reduced, confirming the effectiveness of the experimental stimulation conditions. The minoxidil groups exhibited a remarkably substantial increase in VEGF content, thereby confirming the effectiveness of the positive controls. Furthermore, the NMN groups demonstrated a significant increase in VEGF content compared to the control groups. The VEGF content reached 3771.76 ± 187.47 pg/mL.

### 2.6. NMN Downregulation AR Expression and Activation Wnt/β-Catenin Pathway

To investigate the effects of NMN on the mRNA expression levels of AR genes associated with AGA, qRT-PCR was performed. Primary HDPCs were used as an in vivo model for relevant AGA conditions. The skin’s response to DHT is mainly influenced by androgen receptors (ARs) [29,30,31]. With the increase of cellular levels of DHT, the AR expression also significantly increases [31]. The experimental results validate this correlation, as shown in Figure 5D. The control groups exhibited a significant upregulation in AR gene expression, demonstrating the effectiveness of the stimulation conditions in our tests. Conversely, compared to the control group, the minoxidil groups displayed a noteworthy decrease in AR gene expression, indicating the efficacy of the positive standard controls. Especially, the minoxidil groups demonstrated a 44.52% inhibition rate of AR gene expression compared to the control group. In addition, the NMN groups (0.316 mg/mL) significantly downregulated the expression of the AR gene, resulting in an inhibition rate of 41.78% compared to the control groups.

Wnt signals play a significant role in facilitating intercellular communication between nearby cells. Androgens disrupt the function of Wnt signals by increasing the production of DKK-1, which subsequently affects hair growth. Hence, the measurement of DKK-1 expression can provides some valuable insights into the impact of NMN on hair growth. The results demonstrate a marked elevation in DKK-1 expression in HDPC cultures when treated with DHT compared to BC cultures (Figure 5C). Importantly, minoxidil effectively reduces DKK-1 expression in comparison to the control groups, achieving an inhibition rate of 71.19%. Moreover, the NMN groups also exhibit a significant decrease in DKK-1 expression, with an inhibition rate of 75.16%, surpassing that of the minoxidil groups. Furthermore, the Wnt/β-catenin signaling pathway also plays a crucial role in the development and regeneration of the embryonic hair follicle during the hair cycle. The expression of β-catenin in HDPCs was examined in this study. Compared to the BC groups, the control groups treated with DHT showed a decrease in β-catenin expression, indicating the effectiveness of DHT stimulation (Figure 5D). In contrast, the minoxidil group exhibited a significant increase in β-catenin expression compared to the control group, confirming the efficacy of minoxidil. The NMN groups demonstrated considerably higher expression of β-catenin than the control groups.

### 2.7. Effects of NMN on the Capacity to Eliminate ROS

**Figure 6:**
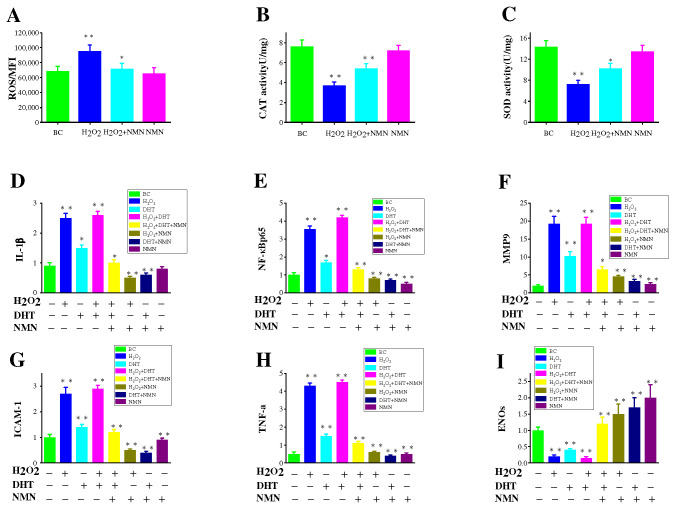
The effect of NMN on free radicals and related inflammatory factors. (**A**) The effect of NMN on free radicals induced by H_2_O_2_. (**B**) The effect of NMN on antioxidant enzymes CAT induced by H_2_O_2_. (**C**) The effect of NMN on antioxidant enzymes SOD induced by H_2_O_2_. (**D**–**I**) Detection of mRNA expression levels of TNF-α, IL-1β, ICAM1, MMP-9, eNOs, and NF-κB p65 using reverse transcription method. The results represent the average of three parallel experiments. Statistical analysis was conducted using the *t*-test, with the BC groups as the reference. Significance levels are expressed as: *p*-value < 0.05 as *, *p*-value < 0.01 as **.

Oxidative stress (OS) is a detrimental consequence caused by free radicals within the body and is considered a significant factor contributing to aging and illness. The oxidative stress resulted from ROS is believed to impair hair papilla cells, thus causing hair loss. Based on the results in Figure 6A, it is evident that NMN can effectively decrease the levels of ROS induced by hydrogen peroxide. In order to study whether NMN’s antioxidant properties enable it to combat cell damage induced by hydrogen peroxide in HDPCs, the functions of CAT and SOD were assessed. As depicted in Figure 6B,C, it can be observed that the activity of CAT and SOD is significantly enhanced by NMN, as opposed to the control group.

### 2.8. Inflammatory Pathways Associated with H_2_O_2_, DHT and NMN

Increased levels of IL-6 have been demonstrated to impact hair growth [12]. Previous results have indicated higher interleukin levels in balding hair dermal papilla cells (HDPCs) from individuals with androgenetic alopecia (AGA) compared to those without hair loss [32]. DHT was administered at a concentration of 800 nM and subsequently treated with 0.3125 mg/mL NMN in HDPCs cultures determines the expression of IL-6 and IL-1β (Figure 5E,F). Notably, significant expression of these factors was observed in hair papilla cells following stimulation with DHT and hydrogen peroxide. In addition, it was discovered that NF-κB p65 expression was significantly increased under the influence of DHT and hydrogen peroxide but could be markedly improved by NMN. NF-κB p65 is recognized as the primary regulatory factor implicated in inflammatory response. To confirm this finding, genes (IL-1β, MMP-9, ICAM-1, TNF-α) associated with inflammatory response were analyzed using reverse transcription. The results in Figure 6D–H indicate that the genes involved in the NF-κB p65 inflammatory response pathway displayed varying degrees of upregulation in comparison to the control group. NMN exhibited the ability to effectively suppress inflammatory factors induced by DHT and H_2_O_2_, while also increasing the expression of eNOs (endothelial nitric oxide synthase). eNOS is considered a gene related to the production of vascular endothelial growth factor (VEGF).

## 3. Discussion

Angiogenesis is regulated by the physiology and pathology of vascular endothelial growth factor (VEGF) [33]. VEGF activity plays a crucial role in hair growth by enhancing the nutrient supply to hair follicles. Several studies have shown that hair follicles affected by androgen alopecia exhibit reduced levels of VEGF [34]. During the anagen phase, there is a significant increase in blood VEGF expression, whereas expression levels are low during the catagen and telogen phases [35]. The expression of VEGF can be stimulated by various herbs and chemicals. For instance, the administration of minoxidil induces higher VEGF expression in human hair dermal papilla cells (HDPCs) [36]. Sinapic acid treatment has been found to increase the proliferation of human hair follicle derived HDPCs and enhance VEGF expression [37]. In vitro studies have demonstrated that *Carthamus tinctorius* L. extract (CTE) can elevate VEGF mRNA expression [33]. Additionally, shikimic acid upregulates VEGF mRNA expression in HDPCs [38]. In this study, a solution of NMN at a concentration of 2.5 mg/mL was added to HDPCs induced by DHT, resulting in a significant elevation of VEGF expression to 3771.76 ± 187.47 pg/mL (Figure 4). This level of stimulation was equivalent to that achieved by minoxidil. Therefore, NMN promotes hair growth by upregulating VEGF expression in HDPCs.

**Figure 7:**
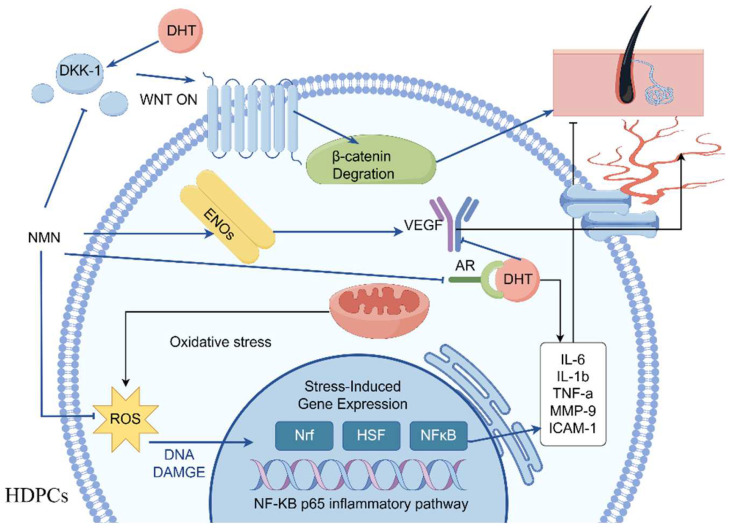
NMN promotes hair growth by regulating the paracrine system of cells. NMN inhibits IL-6 secretion while promoting the upregulation of VEGF and β-catenin gene expression, and simultaneously downregulating the expression of DKK-1 and AR genes. NMN reduces oxidative stress levels by activating antioxidant enzymes, thereby inhibiting the NF-κB p65 inflammatory pathway. The figure was drawn by Figdraw (ID: WYRPAb5f17).

The stimulation results of HDPCs with DHT in an elevation in the expression of inflame matory factors including IL-6, IL-1β and TNF-α. In AGA, HDPCs from balding individuals exhibit higher levels of IL-6 compared to those from non-balding individuals. By inhibiting the growth of stromal cells, IL-6 promotes the degeneration of mouse hair follicles and inhibits the elongation of human hair shafts [16]. HDPCs treated with DHT produce a substantial quantity of IL-6, IL-1β and TNF-α, but its production can be effectively suppressed by NMN. Hydrogen peroxide induced HDPCs model was used to reveal the pathway of NMN reducing inflammatory factors. In hydrogen peroxide induced HDPCs, ROS was highly expressed as shown in Figure 6A, while NMN significantly reduced ROS expression levels compared to the control group. NMN reduced the level of ROS by increasing the activity of CAT and SOD enzymes, thereby reducing the damage caused by oxidative stress to HDPCs, as shown in Figure 6B,C. When oxidative stress occurs, the activation of transcription factor NF-κB p65 causes the activation of cellular inflammatory factors, leading to high expression of IL-1β and TNF-α. MMP-9 and ICAM-1 are two other expression factors in the NF-κB p65 inflammatory pathway, which were also significantly expressed under the stimulation of H_2_O_2_ and DHT, while NMN can significantly lower their expression levels. Therefore, it can be inferred that NMN inhibits the NF-κB p65 inflammatory signaling pathway induced by DHT, thereby promoting hair growth (Figure 7).

AGA is believed to be influenced by AR, a nuclear receptor belonging to the class of receptors that can bind to either ligand or steroid hormones [30]. The action of the DHT androgen alopecia region is mediated through AR. Regarding patients with AGA who experience hair loss, there is abnormal expression of AR in the frontal and vertex regions, but not in non-balding areas. Furthermore, gene association studies have demonstrated a strong correlation between AGA phenotypes and AR [39]. The androgen/AR signal causes damage to the DNA in HDPCs in the balding region, which accelerates premature aging [40]. Hence, DHT may contribute to the miniaturization of AGA hair follicles. The Wnt/β-catenin signal pathway is negatively impacted by ligand-activated AR in the HDPC of individuals with AGA [41]. Therefore, the upregulation of AR induced by DHT plays an important role in androgen alopecia, but NMN can effectively decrease AR expression in HDPCs, potentially contributing to the regulation of hair growth in AGA.

DKK-1 belongs to the Dickkopf protein family, which comprises two cysteine-rich do mains separated by a linker region [42]. It functions as an antagonist of the canonical Wnt signaling pathway by inhibiting the interaction between LRP5/6 and Wnt and by forming a ternary complex with KREMEN, thereby facilitating the internalization of LRP5/6 [43]. Previous results have indicated a correlation between the expression of DKK-1 and male pattern baldness [44]. In our study, the genes regulated by dihydrotestosterone (DHT) in hair loss-associated dermal papilla cells (HDPCs), the gene DKK-1 was discovered to be significantly upregulated. Moreover, treatment of HDPCs with DHT resulted in increased expression of DKK-1 mRNA, which was validated through ELISA analysis. Our research findings support these observations, as depicted in Figure 5C. Additionally, treatment with rhDKK-1 led to reduced hair follicle length in comparison to the control group, whereas treatment with DKK-1 antibodies increased hair follicle length. The primary mechanism by which DKK-1 modulates hair growth involves the blockade of typical Wnt-mediated β-catenin signaling and the induction of the pro-apoptotic protein Bax, leading to apoptosis of keratinocytes in the outer root sheath. In our study, the addition of NMN to DHT-induced HDPCs significantly decreased the expression of DKK-1, similar to the expression observed in the control group treated with minoxidil. The DHT-induced downregulation of DKK-1 expression may represent one of the mechanisms through which NMN mitigates androgenetic alopecia, as implied by the experimental data.

The wnt/β-catenin signaling pathway plays a crucial role in the development and regrowth of hair follicles [20]. One of its functions is to regulate the formation and regrowth of hair by involving outer root sheath cells and HDPCs [45]. Abnormalities in Wnt signal and β-catenin expression can have negative effects on hair growth, as reduced Wnt signaling, and β-catenin expression are associated with hair loss. Studies have demonstrated that inhibiting β-catenin activity hinders the proliferation of neighboring mesenchymal cells [46]. In the cytoplasm, β-catenin binds to the E3-ubiquitin ligase (β-TrCP), which marks it for recognition and subsequent degradation through ubiquitination. Phosphorylation of β-catenin aids in its ubiquitination and subsequent degradation by the proteasome [47]. Suppression of the Wnt signal leads to β-catenin degradation in the cytoplasm through the activity of the proteasome. However, DKK-1 serves as a natural inhibitor of the Wnt signal. Additionally, elevated levels of AR can promote the degradation of β-catenin [48]. In patients with androgenetic alopecia, the expression of AR and DKK-1 in HDPCs within the balding region is higher compared to the non-balding region, inhibiting the Wnt/β-catenin regulatory pathway and resulting in hair thinning. The findings of this study indicate that NMN significantly increases RNA levels of β-catenin compared to HDPCs induced by DHT. This suggests that NMN may suppress the expression of DKK-1 and AR, leading to an elevation in β-catenin expression, which in turn promotes hair growth in mice.

## 4. Materials and Methods 

### 4.1. Materials and Reagents

HDPCs and MSCM were purchased from Guangdong BioCell General Testing Co., Ltd., Dongguan, China. DMSO, dihydrotestosterone (99%), Minoxidil (99%), MTT, NMN (99.5%), and Dexamethasone (99%) were obtained from Sigma-Aldrich, St. Louis, MO, USA. Phosphate-buffered saline (PBS) and Fetal bovine serum (FBS) were obtained from Beijing Solarbio Science & Technology Co., Ltd., Beijing, China. RNAiso Plus, Reverse Transcription System, and fluorescent dye were purchased from Accurate Biology Co., Ltd., Shanghai, China. IL-6 ELISA kits and VEGF ELISA kits were obtained from Abcam (Shanghai) Trading Co., Ltd., Shanghai, China. The microplate reader (Bio-Tek, Epoch, Winooski, VT, USA), CO_2_ incubator (Thermo Fisher Scientific, 1501, Waltham, MA, USA), ordinary PCR (Bosch, Gerlingen, Germany), qPCR (Bio-Rad, CFX-96, Hercules, CA, USA), and an inverted microscope (Olympus, CKX 41, Tokyo, Japan) were used.

### 4.2. Animal Experiments

C57BL/6 mice from the Animal Experiment Center of Sun Yat-Sen University were housed in sterilized polypropylene cages under a 12 h light/dark cycle at a temperature of 23 ± 1 °C and humidity of 35–60%. All mice received equal amounts of food and water, and measures were taken to minimize the pain experienced by the mice. Hair growth-promoting activity was examined in four randomized groups (*n* = 6) of mice. The animal experimental ethical inspection form of Guangxi University (Guangxi, China) was approved as the protocol (No: GXU-2023-006, From 20 March 2023 to 20 July 2023). On the sixth day of the mouse stress test, all the mice underwent hair removal. First, the mice were anesthetized by injecting anesthetic (obtained from Sigma-Aldrich, USA). A mixture of 1:1 rosin and paraffin (from Guangdong BioCell General Testing Co., Ltd., China) were heated with an alcohol lamp until it melted, then cooled slightly. The melted mixture was evenly applied to the mice’s backs using a cotton swab. After the rosin/paraffin mixture solidified, it was carefully removed from the tail of the mouse towards the head, following the direction of hair growth. Any remaining hair was cut off with tweezers. After hair removal, the hair follicles enter a resting period. Next, the mice in the sample group (NMN group), positive control group (Minoxidil group), and negative control group were treated with 5% Na_2_S (Beijing Solarbio Science & Technology Co., Ltd., Beijing, China) warmed to 37 °C in advance) applied to the mice’ backs. After 5 min, the 5% Na_2_S was wiped off with warm water. Once wiped clean, a towel soaked in warm water was applied to the mice’s backs for 2 to 4 min. The blank group (BC group) did not receive any treatment. The drug was administered to the mice’s skin on the second day after depilation. A daily dose of 200 mL of the drug was administered. Before administration, 75% alcohol (Beijing Solarbio Science and Technology Co., Ltd., Beijing, China) was used to disinfect all the mice’s skin samples. The BC group and negative control groups received solvents used in other test groups, positive controls received 5% minoxidil and the NMN groups received 0.5% NMN.

### 4.3. H & E Staining

As previously described, H & E staining was performed [13]. Four groups were in cluded in the evaluation: DHT groups, minoxidil (5%) groups, BC groups, and NMN groups. The tissue samples were fixed by using Bouin’s solution (G-CLONE (Beijing, China) Biotechnology Co., Ltd., Beijing, China), dehydrated with alcohol, and made transparent using xylene (G-CLONE (Beijing) Biotechnology Co., Ltd., China). The transparent tissue was then embedded in melted paraffin (G-CLONE (Beijing) Biotechnology Co., Ltd., China), cooled, and sliced using a slicer. H & E staining was applied to the tissue sections, which were examined under a Nikon-Eclipse Ni-E microscope.

### 4.4. Cell Cultures

Hair dermal papilla cells (HDPCs) were provided by Guangdong BioCell General Testing Co., Ltd., China. Bovine serum was used to culture the HDPCs in 10% (*v*/*v*) medium. They were seeded in a culture flask at a ratio of 1:2 and supple mented with fresh culture medium. The HDPCs were placed in a cell incubator at 37 °C, 5% CO_2_, and saturated humidity for static culture. Fresh medium was added every 2–3 days to the culture medium. HDPCs between passes to 3–10 passages were used for the experiments.

### 4.5. Cytotoxicity Test

The cytotoxicity experiments involved five groups: blank control (BC), solvent con trol (SC), positive control (minoxidil), and sample group (NMN). In the sample group, each sample was tested at eight concentration gradients as 0.0781 mg/mL, 0.1563 mg/mL, 0.3125 mg/mL, 0.625 mg/mL, 1.25 mg/mL 2.5 mg/mL, 5 mg/mL, 10 mg/mL, with three replicate wells set for each concentration gradient.

Drug administration was performed when the cell planking rate in the 96-well plate reached 50~60%. In the SC group, 200 μL of the medium was added per well, while in the PC group, 200 μL 10% DMSO was added per well. The sample group received 200 μL of medium containing the corresponding concentration of NMN in each well. The BC group was inoculated without cells, and only 200 μL of cell culture medium (Guangdong BioCell General Testing Co., Ltd., China) was added. After completion of drug administration, the 96-well plates were incubated at 37 °C, with 5% CO_2_ for 24 h. After 24 h, the supernatant was discarded, and an MTT working solution (0.5 mg/mL) was added to each well. The samples were then incubated for four hours at 37 °C. Following incubation, the supernatant was discarded, and 150 µL DMSO was added to each well. The OD was determined at 490 nm. The relative cell viability was calculated using the following formula:

$$(\mathrm{cell}\;\mathrm{viability})\;\%=\frac{OD\left(SC\right)-OD(BC)}{OD\left(PC\right)-OD(BC)}\times 100\%$$

### 4.6. RNA Preparation and qRT-PCR

Vaccination: Hair papilla cells were seeded at 3.0 × 10^5^ cells/well into 6-well plates and in cubated 24 h in a (37 °C, CO_2_) incubator. DHT stimulation and administration: In the experimental group, DHT stimulation at a concentration of 800 nM was initiated when the cell confluence in the 6-well plate reached approximately 50–60%. Following a 24 h incubation in a (37 °C, CO_2_) incubator, the cell supernatant was collected and washed twice with 1 mL of PBS per well. Subsequently, 1 mL of RNAiso Plus was added to each well, cells were split, and samples were collected for further analysis. RNA was extracted and reversed transcribed into cDNA for fluorescence quantitative PCR detection. 2^−△△CT^ was used to calculate mRNA expression. In the qRT-PCR analysis, Accurate Biology’s SYBR Green I and Bio-Rad’s CFX-96 Touch were employed. A list of primers used in this study is presented in Table 1.

### 4.7. VEGF, IL-6 and IL-1β ELISA Detection

HDCPs were cultured at 3.0 × 10^5^ cells/well into 6-well plates and incubated 24 h in a (37 °C, CO_2_) incubator. In the test group, DHT stimulation at a concentration of 800 nM was applied once the cell confluence in the 6-well plate reached approximately 50–60%. Subsequently, the cells were incubated in a (37 °C, CO_2_) incubator for 24 h. After 24 h culture, 500 mL culture supernatant was collected from the cultured cells. ELISA kits were used to measure VEGF and IL-6 levels in the supernatant (Abcam (Shanghai) Trading Co., Ltd.).

### 4.8. Statistical Analysis

Data visualization and analysis were conducted using GraphPad Prism 9 software. The results are presented as Mean ± SD. Statistical comparisons between groups were performed using two-tailed *t*-tests. A significance level of *p* < 0.05 was considered statistically significant.

## 5. Conclusions

The double-blind experiment revealed that mice exposed to hair removal exhibited a cessation in hair growth. However, the administration of NMN at a specific concentration in the NMN groups stimulated hair growth during the growth phase, similar to the effects observed in the control and blank groups. The effects of NMN were comparable to those of minoxidil. In the in vitro HDPCs experiment, a concentration of 2.5 mg/mL of NMN significantly increased VEGF secretion from HDPCs by 65.19% in comparison to the control groups. Additionally, the IL-6 content notably decreased with an inhibition rate of 13.60%. NMN was observed to downregulate the expression of DKK-1 and AR genes by 75.16% and 41.78%, respectively. Furthermore, there was a significant upregulation of β-catenin expression at a rate of 125.71%. These findings demonstrate that at this concentration, NMN inhibits IL-6 secretion while promoting the upregulation of VEGF and β-catenin gene expression, and simultaneously downregulating the expression of DKK-1 and AR genes. This mechanism contributes to the anti-hair loss effect of NMN, thereby achieving the desired preventative effect. Moreover, the cytotoxicity MTT test revealed that the administered experimental concentration of NMN did not induce any cellular toxicity.

## Figures and Tables

**Table 1:** Primer pairs used for qRT-PCR.

Target	Forward Primer	Reverse Primer
DKK-1	ATGCGTCACGCTATGTGCTG	TGGAATACCCATCCAAGGTGCTA
AR	CTCTCACATGTGGAAGCTGCAAG	TTTCCGAAGACGACAAGATGGAC
β-catenin	TTAGCTGGTGGGCTGCAGAA	GGGTCCACCACTAGCCAGTATGA
ICAM-1	CTGAAAGATGAGCTCGAGAGT	AAACGAATACACGGTGATGGTA
IL-1β	TCGCAGCAGCACATCAACAAGAG	TGCTCATGTCCTCATCCTGGAAGG
MMP-9	CAAAGACCTGAAAACCTCCAAC	GACTGCTTCTCTCCCATCATC
eNOs	CTGAGAGCCTGCAATTACTACC	TTTCCACAGAGAGGATTGTAGC
TNF-α	ATGTCTCAGCCTCTTCTCATTC	GCTTGTCACTCGAATTTTGAGA
NF-κB p65	TCGAGTCTCCATGCAGCTACGG	CGGTGGCGATCATCTGTGTCTG

## Data Availability

Data available on request due to restrictions eg privacy or ethical.
